# Production and reproduction responses for dairy cattle supplemented with oral calcium bolus after calving: Systematic review and meta-analysis

**DOI:** 10.3168/jdsc.2022-0235

**Published:** 2022-11-18

**Authors:** Ainhoa Valldecabres, Rúbia Branco-Lopes, Christian Bernal-Córdoba, Noelia Silva-del-Río

**Affiliations:** 1 Teagasc, Animal and Grassland Research and Innovation Center, Moorepark, Fermoy, Co. Cork, Ireland P61 C996; 2 School of Veterinary Medicine, Department of Population Health and Reproduction, University of California, Davis 95616; 3 Veterinary Medicine Teaching and Research Center, Tulare, CA 93274

## Abstract

Among the commonly implemented strategies developed to support calcium (Ca) metabolism postpartum, oral Ca supplementation is within the most studied. However, research shows varying responses to treatment in regard to production and reproduction. Our objectives were (1) to identify and synthesize the literature evaluating the associations between postpartum oral Ca supplementation as bolus and milk yield and risk of pregnancy to first service using a systematic review, and (2) to quantify these associations using meta-analytical methods. Nine relevant studies published between January 2010 and September 2021 were identified after systematic search of 4 databases (Biosis, CAB Abstracts, Medline, Scopus). The studies were conducted in commercial confined and grazing farms. Eight of the 9 studies reported feeding low to negative dietary cation-anion difference diets prepartum. Oral Ca bolus supplementation strategies varied among studies but were predominantly centered on the first 24 h postpartum. Milk yield and pregnancy to first service were evaluated in 9 and 6 of the studies, respectively. Other productive (energy-corrected milk yield, peak milk yield, or fat and protein concentrations) and reproductive (estrus cyclicity, days from calving to first service, or pregnancy by 150 or 210 days in milk) outcomes were evaluated in 4 of the studies. The meta-analyses revealed a lack of evidence for associations between prophylactic blanket postpartum oral Ca bolus supplementation and milk yield (including 8 studies) or risk of pregnancy to first service (including 6 studies). Some of the evaluated studies reported statistically significant associations after data stratification. However, not enough studies reported estimates for the evaluated conditional factors to perform a meta-analysis among the identified subgroups. In conclusion, future research should study and report the production, reproduction, and health estimates of cow-level conditional factors of interest for the evaluated response, and investigate alternative regimes of oral Ca bolus supplementation.

## Highlights

• Oral Ca bolus supplementation was not associated with milk yield or pregnancy to first service.
• Supplementation protocols must be reevaluated if group level effects are sought.
• Further research is needed to evaluate target oral Ca bolus supplementation.

## Graphical Abstract

**Figure:**
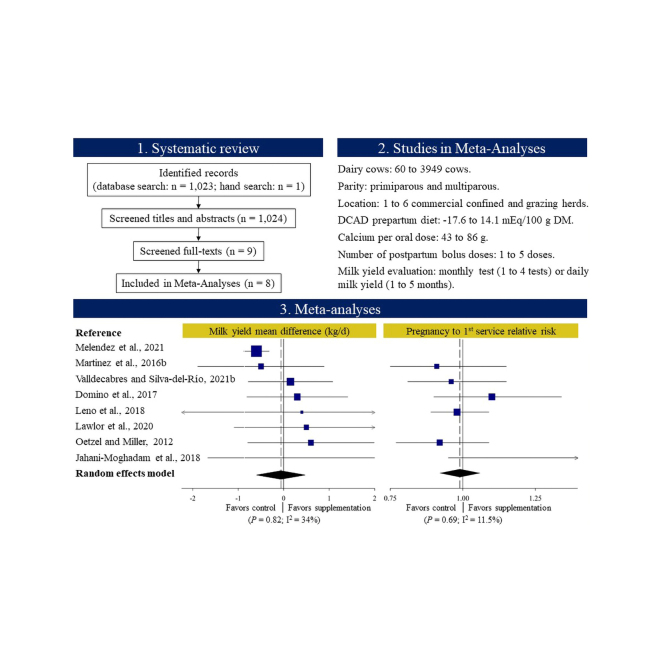
**Summary:** Meta-analytical methods were used to quantify the associations between prophylactic blanket postpartum oral Ca bolus supplementation and milk yield and risk of pregnancy to first service in dairy cows. Nine relevant studies were identified following a systematic review of the literature published between January 2010 and September 2021. Eight of the eligible studies reported feeding low or negative dietary cation-anion difference diets prepartum. The meta-analyses revealed a lack of evidence for a group level response on milk yield or risk of pregnancy to first service after prophylactic blanket postpartum oral Ca bolus supplementation. Future research should study and report the production, reproduction, and health estimates of cow-level conditional factors of interest for the evaluated response, and investigate alternative regimens of oral Ca bolus supplementation.

Processes such as colostrogenesis, milk synthesis, immune activation, or inflammation challenge the homeostatic and homeorhetic mechanisms responsible to maintain blood calcium (Ca) concentration, and may lead to low blood Ca concentration during the peripartum period (Horst et al., 2020; Valldecabres and Silva-del-Río, 2022). Low blood Ca concentration postpartum has been associated in epidemiological studies with undesired outcomes such as increased risk of diseases and reproductive inefficiency (Rodríguez et al., 2017; Valldecabres and Silva-del-Río, 2021a). Thus, management strategies aiming to support blood Ca concentration during the periparturient period are common in commercial farms. Based on US national statistics (USDA-NAHMS, 2014), postpartum Ca supplementation (intravenous, subcutaneous, or oral) is provided in 69% of the farms. Oral Ca supplementation (liquid, gel, paste, or bolus form) is based on the premise that large amounts of soluble forms of Ca create a concentration gradient between the digestive tract and the extracellular fluid, which favors the passive absorption of Ca (Goff, 2018). In the last decade, studies have been carried out to evaluate oral Ca bolus supplementation following the guidelines suggested by commercial brands (2 bolus doses: one immediately after calving and another 12–24 h later), and single or additional oral Ca doses (Martinez et al., 2016b; Leno et al., 2018; Valldecabres and Silva-del-Río, 2021b). Oral Ca supplementation on the form of CaCl_2_ and CaSO_4_ as boluses is usually successful in increasing blood Ca concentration within 1 h after supplementation; however, after 48 h blood Ca concentration is similar between supplemented and control cows regardless of the dose of Ca administered and the duration of the administration (Martinez et al., 2016a; Valldecabres et al., 2018). Nevertheless, at the farm level, interest is reliant on its application to improve production, reproduction, or health.

Systematic reviews and meta-analysis are powerful synthesis methods that can be used to evaluate the existing evidence on the efficacy of oral Ca bolus supplementation from published randomized controlled trials (O'Connor et al., 2014a,b). The objectives of this study were (1) to identify and synthesize the literature evaluating the associations between postpartum oral Ca bolus supplementation and milk yield and risk of pregnancy to first service using a systematic review, and (2) to quantify these associations using meta-analytical methods.

The systematic review and meta-analysis protocol was deposited with the University of California-Davis repository (https://escholarship.org/uc/item/0h5330hv; Valldecabres et al., 2021) in August 2021, and later modified to include only manuscripts published from January 2010. The literature search, designed by an experienced librarian following PICOS (population, intervention, comparison, study design), was conducted in September 2021 using 4 databases: Biosis (Web of Science), CAB Abstracts (CAB Direct), Medline (PubMed), and Scopus (Scopus). Thus, the literature search was framed between January 2010 and September 2021. After a pilot test, titles and abstracts of the retrieved publications were independently screened by 2 reviewers using the following screening questions: Does the title or abstract describe (1) a study involving dairy cows supplemented with oral Ca bolus postpartum, (2) a primary intervention study using negative controls, (3) effects on milk yield and pregnancy to first service, and (4) oral Ca supplementation as a prophylactic strategy (not treatment for sick animals)? If both reviewers answered yes to all these questions, the full text was screened. The first author extracted population, intervention, and comparator information, and the 2 reviewers manually extracted outcomes data from the studies [means, SD, SE, risk ratios (**RR**), 95% CI; Sargeant and O'Connor, 2014]. Authors from 3 manuscripts were contacted due to incomplete data reporting in the published manuscript. Overall group level estimates and associated measures of variability for milk yield were obtained for 2 manuscripts (Leno et al., 2018; Lawlor et al., 2020). However, the exact number of cows allocated to each treatment could not be obtained from one study after authors were contacted (Jahani-Moghadam et al., 2020). Conflicts between the 2 reviewers were discussed until a consensus was reached.

Meta-analyses were performed in R 4.0.3 (R Foundation for Statistical Computing) using RStudio version 1.3.1093 (RStudio Inc.) with meta package (Schwarzer, 2007) to obtain a single summary estimate of the oral Ca bolus supplementation effect on milk yield and risk of pregnancy to first service. Mean difference (**MD**; milk yield) and RR (pregnancy to first service) were computed with their respective within-study variance (95% CI). Both meta-analyses were performed with a random effects model, using the generic inverse variance method to weight the studies and the restricted maximum likelihood (REML) method to estimate the between-study variance. Heterogeneity between studies was assessed with the I^2^ statistic (Higgins and Thompson, 2002).

The database search strategy identified 1,023 studies. After the title and abstract screening, 8 articles were selected for full text screening. Additionally, 1 study was identified after hand search. A description of the study population is reported in Table 1. Selected studies were conducted in commercial confined (n = 7) and grazing (n = 2) farms located in the United States (n = 5), Iran (n = 2), Chile (n = 1), and Ireland (n = 1). The reported fed DCAD was low to negative (≤14 mEq/100 g of DM) and diets provided varying Ca concentrations prepartum (Table 1; n = 8). One study did not report prepartum diet composition. The number of herds enrolled in each of the selected studies was 1 (n = 6), 2 (n = 2), or 6 (n = 1). Sample size was based on the ability to detect differences in serum Ca concentration (n = 2), milk yield (n = 2), milk yield and pregnancy to first service (n = 1), or not justified (n = 4). One (n = 8) or 2 (n = 1) postpartum oral Ca supplementation strategies involving 1 (n = 1), 2 (n = 7), or 5 doses (n = 1), predominantly of CaCl_2_, were evaluated in the studies (Table 2). Treatment allocation was performed by random assignment of the first cow enrolled in the study and subsequent sequential treatments assignment (n = 3), by random assignment of each cow (n = 5), or not reported (n = 1). Administration of treatments was performed by researchers (n = 2), farm personnel (n = 3), or not described (n = 4; Table 2). Milk yield was evaluated based on monthly test [4 tests (n = 1), 3 tests (n = 2), or 1 test (n = 1)] or daily milk yield data [20 d (n = 1), 28 d (n = 1), 30 d (n = 1), 30 d and 5 mo (n = 1), 10 wk (n = 1), 90 d (n = 1)], using data from milk recording associations (n = 3), on-farm software (n = 3), both sources (n = 1), or not specified (n = 2). Additional production outcomes were evaluated in some studies: peak milk yield (n = 1), ECM yield (n = 2), FCM yield (n = 2), and milk fat and protein concentrations (n = 4) and yields (n = 3). Pregnancy to first service was evaluated in 6 of the 9 studies, using data obtained from the record-keeping herd management software (n = 3), by the researchers (n = 1), or from no reported origin (n = 2). Other reproductive outcomes evaluated included estrus cyclicity (n = 1), days from calving to first service (n = 1), and pregnancy by 150 (n = 3), 180 (n = 1) or 210 DIM (n = 1). Health outcomes were also reported in some of the studies including clinical hypocalcemia (n = 3), subclinical hypocalcemia (n = 4), ketosis or BHB concentrations (n = 4), retained placenta (n = 4), displaced abomasum (n = 3), metritis (n = 3), endometritis (n = 1), and mastitis (n = 3).

**Table 1:** Description of study population from the 9 eligible studies identified in the systematic review evaluating the association of oral Ca bolus supplementation with production and reproduction outcomes^1^

Reference	Cows (n)	Parity	Breed	Herds (n)	Prepartum diet	
					DCAD (mEq/100 g of DM)	Ca (% of DM)
Domino et al. (2017)	998	M	HO	1	−15 to −10	NR
Jahani-Moghadam et al. (2018)	66	M	HO	1	8.9	0.5
Jahani-Moghadam et al. (2020)^2^	24	M	HO	1	−9.4	1.3
Lawlor et al. (2020)	60	M	NR	1	NR	NR
Leno et al. (2018)	2,962	M^3^	HO	6	−6.9 to 14.1	0.86 to 1.78
Martinez et al. (2016b)	444	P, M^4^	HO	1	−15.3 to 0.6	1.02 to 1.23
Melendez et al. (2021)	60	M	HO	1	−8.6	0.84
Oetzel and Miller (2012)	927	M	HO	2	−10.9 to −1.8	0.88 to 0.93
Valldecabres and Silva-del-Río (2021b)	1,129	M	JE, JE × HO	2	−17.6 to −16.8	2.46 to 2.86

1 P = primiparous postpartum; M = multiparous postpartum; HO = Holstein; JE = Jersey; NR = not reported.

2 Study was not included in the final meta-analysis due to incomplete reporting.

3 Primiparous cows were also included in the study (n = 987). Only data from multiparous cows were extracted for this study.

4 Primiparous and multiparous cows were modeled together. Extracted data correspond to both parity groups.

**Table 2:** Description of oral Ca supplementation treatments from the 9 eligible studies identified in the systematic review evaluating the association of oral Ca bolus supplementation with production and reproduction outcomes

Reference	Treatments: g of Ca (time postpartum)	Ca source
Domino et al. (2017)^1^	43 g (30 min) + 43 g (19 h)^2^	CaCl_2_, CaSO_4_
Jahani-Moghadam et al. (2018)	45 g (calving) + 45 g (24 h)^3^	CaCl_2_, calcium propionate, calcium fumarate
Jahani-Moghadam et al. (2020)^4^	45 g (calving) + 45 g (24 h)^3^	CaCl_2_, calcium propionate, calcium fumarate
Lawlor et al. (2020)^1^	<45 g (<4 h) + < 45 g (8 to 24 h)^3^	CaCl_2_, CaSO_4_^5^
Leno et al. (2018)^1^	54–64 g (<24 h)^2^	CaCl_2_, CaSO_4_, calcium propionate, calcium lactate
Martinez et al. (2016b)	A: 86 g (0 d) + 86 g (1 d)	CaCl_2_, CaSO_4_
	B: A + 43 g (2 d) + 43 g (3 d) + 43 g (4 d)	
Melendez et al. (2021)	44 g (calving) + 44 g (24 h)^3^	CaCl_2_
Oetzel and Miller (2012)^1^	43 g (<2 h) + 43 g (8 to 35 h)^2^	CaCl_2_, CaSO_4_
Valldecabres and Silva-del-Río (2021b)^1^	50–60 g (45 min to 5 h) + 50–60 g (24 to 34 h)	CaCl_2_, CaSO_4_, calcium propionate, calcium lactate

1 Study totally or partially funded by an oral Ca bolus commercial company.

2 Treatments administered by farm personnel.

3 Treatment administrators not specified.

4 Study was not included in the final meta-analysis due to incomplete reporting.

5 Data provided by authors upon request.

Selected studies included only multiparous (n = 7) or primiparous and multiparous cows (n = 2). The studies including primiparous and multiparous cows analyzed data separately (n = 1; Leno et al., 2018) or in combination (n = 1; Martinez et al., 2016b). Estimates were extracted from the former cited studies and separate meta-analyses were conducted of studies including primiparous and multiparous cows (**MA-ParityAll**; 9 eligible studies) and excluding primiparous (**MA-Multiparous**; 8 eligible studies). Also of note, the study by Martinez et al. (2016b) combined the effects of oral Ca bolus supplementation during the first 2 d postpartum, with those of oral Ca bolus supplementation during the first 4 d postpartum for the group level comparison after nonsignificant effect of oral Ca supplementation length. Thus, MA-Multiparous only includes multiparous cows and studies providing oral Ca bolus supplementation within the first 2 d postpartum. For the association between milk yield and oral Ca supplementation, group level estimates, measure of variability, and number of cows per treatment group were not provided in Leno et al. (2018), Lawlor et al. (2020), and Jahani-Moghadam et al. (2020), respectively. Corresponding authors were contacted, but this information was obtained only from Leno et al. (2018) and Lawlor et al. (2020). Thus, milk yield meta-analyses included 8 (MA-ParityAll) and 7 (MA-Multiparous) studies, and pregnancy to first service meta-analyses included 6 (MA-ParityAll) and 5 (MA-Multiparous) studies. Eligible studies included in the MA evaluated conditional factors for the treatment association with milk yield such as parity [second, third, and ≥fourth (n = 4); second and ≥third (n = 2); primiparous and multiparous (n = 1)], previous lactation milk yield (relative to herd average; n = 5), previous lactation length (n = 2), dry period length (n = 3), gestation length (n = 3), BCS [pre-fresh (n = 1); at calving (n = 2); between 0 and 10 DIM (n = 1)], locomotion score [pre-fresh (n = 1); at calving (n = 1); between 0 and 10 DIM (n = 1)], age at first calving (primiparous; n = 1), calving season/month (n = 2), calving ease (n = 3), twinning (n = 1), stillbirth (n = 1), calf sex (n = 1), risk of developing metritis (n = 1), blood Ca before oral Ca supplementation [categorical (n = 1); continuous (n = 1)], and lameness pre-freshening along with high previous lactation milk yield (n = 1). The same conditional factors were evaluated for the treatment association with pregnancy to first service effect except for previous lactation milk yield (relative to herd average; n = 3), previous lactation days open (n = 1), DIM at first service (n = 2), and breeding strategy (timed AI and heat breeding; n = 3). No cow-level conditional factors were considered in statistical models from 2 studies. Overall, only 3 conditional factors were evaluated in at least 3 of the eligible studies; however, estimates at the oral Ca supplementation group level were only provided when statistically significant interactions between these variables and treatment were observed [previous lactation milk yield (n = 2); gestation length (n = 1); dry period length (n = 0)]. Thus, an insufficient number of studies were available to quantify treatment effects on the identified subpopulations with meta-analytical methods (Jackson and Turner, 2017).

Results from MA-ParityAll showed no evidence of association between prophylactic postpartum blanket oral Ca bolus supplementation and milk yield (8 studies; Figure 1 A; *P* = 0.82) or pregnancy to first service at the group level (6 studies; Figure 1B; *P* = 0.69). Heterogeneity among studies for the association between oral Ca supplementation and milk yield or pregnancy to first service was 33.9 and 11.5%, respectively (*P* = 0.16 and 0.34, respectively). Similarly, results from MA-Multiparous showed no evidence of an association between prophylactic postpartum blanket oral Ca bolus supplementation and milk yield [7 studies; *P* = 0.91; I^2^ = 43.3% (*P* = 0.10)] or pregnancy to first service at the group level [5 studies; *P* = 0.87; I^2^ = 22.3% (*P* = 0.27)].

**Figure 1:**
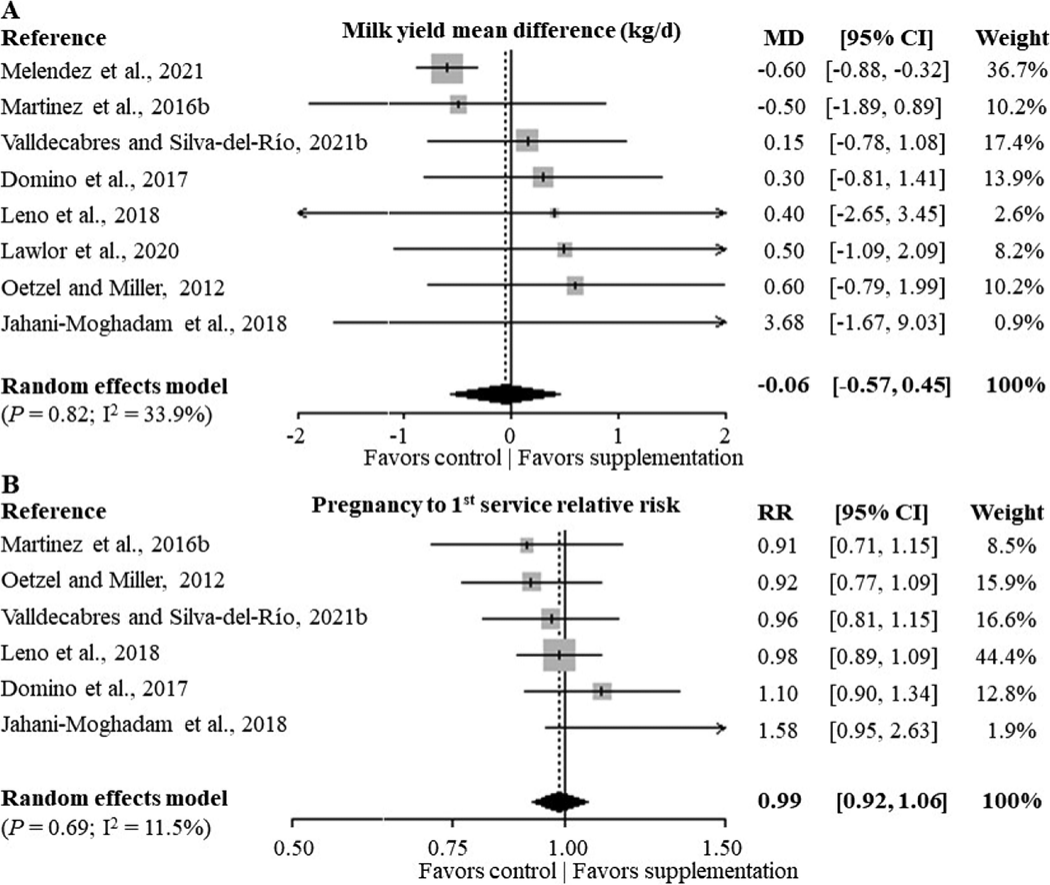
Forest plots of the milk yield mean difference (MD; panel A), pregnancy to first service risk ratio (RR; panel B), and overall summary effect size of prophylactic blanket postpartum oral Ca bolus supplementation in dairy cows. Selected studies included in the meta-analysis enrolled both primiparous and multiparous cows (MA-ParityAll). The solid vertical line represents a MD of zero or no effect; points to the left of the line represent a reduction in milk yield or risk of pregnancy to first service, whereas points to the right of the line indicate an increase. Each square around the point effect represents the effect size [MD (kg/d) or risk ratio] for that study and reflects the relative weight of the study to the overall effect size estimate. The weight that each comparison contributed is in the right-hand column. Left and right limits of the horizontal lines represent the lower and upper 95% CI of the effects size. The bottom diamonds represent overall summary effect size and 95% CI pooled using random effects models. I^2^ statistic (Higgins and Thompson, 2002).

The collective evidence summarized herein suggests that prophylactic blanket oral Ca bolus supplementation during early postpartum is not associated with overall effects on milk yield or pregnancy to first service. Data stratification by parity (2, 3, and ≥4), previous lactation 305-d mature equivalent milk yield, previous lactation length, gestation length, or BCS at calving in some of the selected studies, led to the detection of statistical differences on milk yield between control and oral Ca bolus supplemented cows within subgroups of cows. However, although some conditional factors were evaluated in different studies, estimates by group were only reported when a significant interaction with treatment was detected in the final multivariable models, and thus meta-analyses could not be conducted for the association of oral Ca bolus supplementation and milk yield conditional to the aforementioned variables. Similarly, data stratification by parity (primiparous vs. multiparous) led to the detection of statistically significant positive and negative effects of oral Ca supplementation on pregnancy to first service in one study.

Selected studies evaluated supplementation strategies that targeted cows within the first 48 h postpartum, except one study that evaluated supplementation up to 4 d. These strategies aimed to prevent the blood Ca nadir that most cows reach within 24 h postpartum (Martinez et al., 2016a). A short-lived increase in Ca after treatment administration was reported by 3 eligible studies (Martinez et al., 2016a; Domino et al., 2017; Valldecabres et al., 2018). However, recent research suggest that low blood Ca concentration is only associated with future cows' milk production if calcemia is not restored by 2 to 4 d postpartum (McArt and Neves, 2020). Thus, it is plausible that oral Ca supplementation strategies targeting the first 24 h blood Ca concentration nadir are not the optimum if positive effects on production are the aim. Conversely, epidemiological studies agree on a negative association between low blood Ca levels early postpartum and reproduction (Rodríguez et al., 2017; Umaña Sedó et al., 2018; Valldecabres and Silva-del-Río, 2021a). Nevertheless, Horst et al. (2021) suggest that low blood Ca levels postpartum may reflect normal homeorhetic adjustments for high-producing cows or immune activation. Hence, it is uncertain if they are directly associated with reproduction performance as lactation advances.

Some of the studies summarized herein (Martinez et al., 2016b; Leno et al., 2018; Melendez et al., 2021), reported negative effects of oral Ca bolus supplementation on milk production for cows with lower production potential and cows of second parity, and on reproduction for primiparous and cows with shorter dry periods. This, along with the risk of injury during administration and observed positive results being limited to certain subpopulations of cows, emphasizes the importance of a targeted oral Ca bolus supplementation strategy to maximize profit and prevent unwanted effects of oral Ca supplementation (Gomez et al., 2019). However, there is a lack of consensus on the identification of these subpopulations, and the heterogeneity on the evaluated conditional factors and the subgroups identified in the selected studies has prevented us from conducting meta-analyses to quantify the effect of postpartum oral Ca bolus supplementation among these subgroups and provide specific recommendations for oral Ca bolus supplementation.

It should be noted that most of the studies included in the present meta-analyses were conducted in commercial farms where nutritional strategies aiming to support blood Ca concentration during the peripartum were already in place (low to negative DCAD prepartum diet). Thus, it is uncertain if the conclusions drawn from the present study would extend to postpartum oral Ca bolus supplementation when different prepartum nutritional strategies are implemented (high DCAD). Furthermore, due to the specificity of our research question and incomplete data reporting in some studies, our meta-analyses included a limited number of studies. It is also noteworthy that the small SEM reported by Melendez et al. (2021) and the large SD provided upon request by Leno et al. (2018) led to the milk yield meta-analyses assigning the highest and smallest weight to the smallest and largest study, respectively. A strength of the present systematic review and meta-analysis is the methods. A protocol was developed a priori, reported in accordance with Preferred Reporting Items for Systematic Reviews and Meta-Analyses Protocols (PRISMA-P; Moher et al., 2015), and adhered to the guidelines for systematic review in animal agriculture and veterinary medicine (O'Connor et al., 2014a,b). The search strategy was designed with the support of an experienced librarian and implemented in 4 different databases to increase the likelihood of identifying relevant articles. Additionally, the screening of titles and abstracts and data extraction of outcomes was performed independently by 2 reviewers (A.V. and C.B.C.).

In conclusion, the absence of prophylactic blanket postpartum oral Ca bolus supplementation group level effects on milk yield and risk of pregnancy to first service suggest that oral Ca supplementation protocols may need re-evaluation if group level positive effects on production or reproduction are sought on cows fed low to negative DCAD prepartum diet. At the present, there is an insufficient number of studies reporting estimates for conditional factors of interest or evaluating disease incidence at the group level. If enough eligible studies become available, future meta-analyses should quantify the aforementioned associations, as it may justify the implementation of the supplementation strategies evaluated herein, despite the absence of overall effects on milk yield and pregnancy to first service.
